# Locus-aware decomposition of gene trees with respect to polytomous species trees

**DOI:** 10.1186/s13015-018-0128-1

**Published:** 2018-06-04

**Authors:** Michał Aleksander Ciach, Anna Muszewska, Paweł Górecki

**Affiliations:** 1 Faculty of Mathematics, Informatics and Mechanics, University of Warsaw, Banacha 2, Warsaw, Poland; 2 Institute of Biochemistry and Biophysics, Polish Academy of Sciences, Pawińskiego 5A, Warsaw, Poland

**Keywords:** Rank, Taxon, Ranked species tree, Speciation, Gene duplication, Gene loss, Horizontal gene transfer

## Abstract

**Background:**

Horizontal gene transfer (HGT), a process of acquisition and fixation of foreign genetic material, is an important biological phenomenon. Several approaches to HGT inference have been proposed. However, most of them either rely on approximate, non-phylogenetic methods or on the tree reconciliation, which is computationally intensive and sensitive to parameter values.

**Results:**

We investigate the locus tree inference problem as a possible alternative that combines the advantages of both approaches. We present several algorithms to solve the problem in the parsimony framework. We introduce a novel tree mapping, which allows us to obtain a heuristic solution to the problems of locus tree inference and duplication classification.

**Conclusions:**

Our approach allows for faster comparisons of gene and species trees and improves known algorithms for duplication inference in the presence of polytomies in the species trees. We have implemented our algorithms in a software tool available at https://github.com/mciach/LocusTreeInference.

## Background

Horizontal gene transfer (HGT) is the process of acquisition and fixation of foreign genetic material. It can lead to substantial changes in the ecology and evolution of recipient organism, sometimes leading to the emergence of new pathogens [1]. HGT is interesting both from biological and computational perspective. Several methods of detecting horizontally transferred genes have been proposed, which can be roughly divided into two categories [2]. So-called *surrogate methods* are computationally efficient, yet often imprecise. The other group consists of the *phylogenetic methods*, most notably the tree reconciliation [3].

HGT and gene duplication are examples of evolutionary events in which an organism gains a new locus (referred later on as *locus gain events*). A *locus* is a fragment of a chromosome with a specific gene. The locus gain events cause an *incongruence* between a gene tree and a species tree. A species tree is a schematic representation of an evolutionary history of a set of species, in which a node corresponds to a speciation event (i.e. separation of a group of organisms into two distinct species). Likewise, a gene tree is a schematic representation of the evolutionary history of a set of genes. A node of a gene tree corresponds to an emergence of a new gene, whether due to a speciation or an evolutionary event like HGT or duplication. The leafs of the gene tree are usually labelled by the leafs of the corresponding species tree. Consequently, while the leaf labels of the species trees are unique, a single label may occur multiple times in the gene tree.

Apart from the aforementioned evolutionary events, populational effects are a major source of incongruence between gene and species trees. These effects arise due to the fact that each gene may undergo a mutation, which creates a new nucleotide sequence referred to as an *allele* of the gene. It follows that the set of alleles in a single species can itselt exhibit a complex evolutionary relationship. During a speciation, the alleles present in the population are sorted into two sets corresponding to the diverging populations (a process termed *allele* or *lineage sorting*). It follows that the evolutionary distance between alleles may not correspond to the evolutionary distance between their respective species, causing an incongruence between the species tree and the gene tree inferred from the nucleotide sequences of those alleles. Such incongruence is referred to as an *incomplete lineage sorting* (ILS). It can be shown that as the time between speciations increases, the probability of an ILS decreases exponentially. Therefore, if all the speciations in the considered species tree are separated by a sufficiently large period of time, the populational effects may be considered negligible. For a more detailed discussion of the ILS, the reader is referred to e.g. [4–6].

The new locus created by one of the aforementioned evolutionary events evolves more or less independently of other loci. This observation leads to the concept of a *locus tree* [4, 7], which serves as a schematic represenation of the evolutionary relationship between different loci. A node of a locus tree corresponds to an emergence of a new locus due to either a speciation or an evolutionary event (note that a mutation of the nucleotide sequence does not create a new locus). The loci are assumed to evolve within the branches of the species tree (or, in case of an HGT, between two branches), while the alleles are assumed to evolve within the branches of the locus tree. Therefore, a locus tree is an intermediate concept between the gene and the species tree, as it encompasses the evolutionary events like gene duplication or HGT, but not the populational effects. Another distinction between the gene and locus trees is that the former can be inferred from the nucleotide sequences, while the latter usually needs to be inferred by comparing the gene tree to the species tree.

**Fig. 1:**
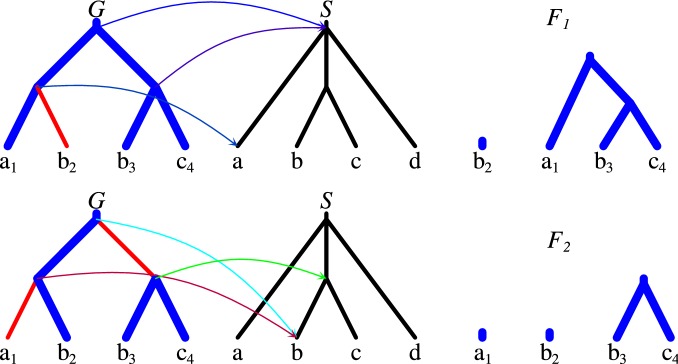
An example of locus trees for $G=((a_1,b_2),(b_3,c_4))$ with two decompositions $F_1$ and $F_2$ consistent with $S=(a, (b, c),d)$. These decompositions are created by cuts indicated with red color. $M^X :G \rightarrow S$ is shown for every set of cuts *X* (for internal nodes). Here, $\Lambda (F_1,S)=\Lambda (F_2,S)=0$, $\Delta (F_1)=$ $2\cdot \mathbf {GAIN}$ and $\Delta (F_2)=3\cdot \mathbf {GAIN}$, i.e., $F_1$ is optimal

When population effects are negligible due to long times between speciations, the evolutionary events are the only source of incongruence between the gene and the species tree. In this case, the evolutionary relationship between loci can be regarded as identical to the evolutionary relationship between alleles. Consequently, the locus tree can be represented as the gene tree with nodes labeled either as speciations or locus gains. In this approach, a *locus gain node* corresponds to the time point in which a new locus is first observed (note that after a duplication, the choice of the “new” versus the “old” locus is often arbitrary). Such labeling allows to decompose the gene tree into a forest of trees representing independent evolutionary histories of different loci by detaching the locus gain nodes (see e.g. Fig. 1). The concept of gene tree decomposition has been investigated earlier in the context of tree comparison [8], but, to our knowledge, not in the context of inference of evolutionary events or locus trees.

Distinguishing between different locus gain events is challenging, as their effects on gene trees are topologically similar. In reconciliation, weights of events have to be specified; these are, however, rarely known. The fact that the results depend strongly on those unknown parameters may undermine the credibility of biological conclusions. To properly estimate the weights, high-quality training datasets are needed, in which inferred events are biologically supported.

Many cases of HGT were found by manual inspection of incongruences in gene trees [9]. Inferring a locus tree facilitates such analyses, as it allows to automatically detect the incongruences. This approach has several advantages over reconciliation. It allows restricting to only two parameters: the locus gain and the locus loss weight. It is also more robust to imprecise data, as improperly placed branches will only be locally detected as new loci, without interfering with the global evolutionary scenario. This allows to disregard the noise when analyzing the tree, and instead focus on several chosen events. The locus tree inference has been addressed in populational genetics setting [4]. However, this approach requires several parameters, like speciation times or population sizes, which are often challenging to obtain. It has also been addressed in parsimony framework in the model of gene duplication and loss [7].

*Our contribution* In this work, we address the problem of locus tree inference when populational effects are negligible. This allows addressing the locus tree inference problem in a parsimony framework, and to adapt a more general approach than presented in [7]. We assume that incongruence between gene and species trees can be caused by locus acquisition events of any kind, including duplications and horizontal gene transfers. We propose to solve the locus tree inference problem by decomposing a binary gene tree into a forest of subtrees that can be embedded into a possibly polytomic species tree, in a way that minimizes the weighted sum of the forest size and the number of loss events. We propose two variants of the problem: the *Locus Tree Inference*, *LTI*, in which forest elements are subtrees of the species tree, and the *Conditional Locus Tree Inference*, *CLTI*, where each forest element is a subtree of some binarization (full refinement) of the species tree. We show a dynamic programming algorithm that solves LTI in *O*(|*G*||*S*|*m*) time and *O*(|*G*||*S*|) space, where *m* is the maximal degree of a node from the species tree. To solve CLTI, we propose a new mapping, called the highest separating rank. Based on the mapping, we show an $O(d|G|+|S|)$ time and $O(|G|+|S|)$ space algorithm, where *d* is the height of *S*, for inferring required and conditional duplications in gene trees, which improves an $O(|G|(d+m)+|S|)$ time solution from [10]. Finally, we propose an efficient heuristic to solve CLTI, and present a comparative study on simulated and empirical data.

## Methods

### Definitions

Let $T=\langle V_T,E_T \rangle$ be a rooted directed tree. For $a, b\in V_T$, by $\mathop {\mathrm {lca}}_T(a,b)$ we denote the lowest common ancestor of *a* and *b* in *T*. We also use the binary order relation $a \preceq b$ if *b* is a node on the path between *a* and the root of *T* (note that $a \preceq a$). Two nodes *a* and *b* are called *siblings*, which is denoted $a={{\mathrm{\mathsf {sibling}}}}(b)$, if they are children of $\mathop {\mathrm {lca}}_T(a,b).$ We call *a* and *b* *comparable* if $a \preceq b$ or $b \preceq a$, otherwise *a* and *b* are called *incomparable*. The parent of a node *a* is denoted as $\mathsf {parent}(a)$. The subtree of *T* rooted at *v* is denoted by *T*(*v*). By *L*(*T*) we denote the set of all leaves in a tree *T* and we use *L*(*v*) instead of *L*(*T*(*v*)). By ${{\mathrm{\mathsf {root}}}}(T)$ we denote the root of tree *T*. A *species tree* *S* is a rooted directed tree in which nodes are called *taxa*. A *gene tree* *G* is a rooted directed binary tree, such that every leaf of *G* is labeled by a leaf-taxon from *S*, i.e., an element of *L*(*S*). Note that a gene tree can have multiple copies of taxons (see Fig. 1). For a node *g* in *G*, by $\mathop {\mathrm {tax}}(g) \subset L(S)$ we denote the set of all labels of leaves from *L*(*g*).

The *lowest common ancestor mapping*, or lca-mapping, between *G* and *S* is a function $M :V_G \rightarrow V_S$ such that $M(g)=t$ if *g* is a leaf labelled by the leaf-taxon *t*, or $M(g)=\mathop {\mathrm {lca}}_S(M(g_1),M(g_2))$ if *g* has two children $g_1$ and $g_2$. An internal node *g* in *G* is a *duplication* if $M(g)=M(g_i)$ for any child $g_i$ of *g*. Every other node, i.e., a leaf or an internal node satisfying $M(g) \succ M(g_i)$ for every child $g_i$ of *g*, is called a *speciation* [11–13].

**Fig. 2:**
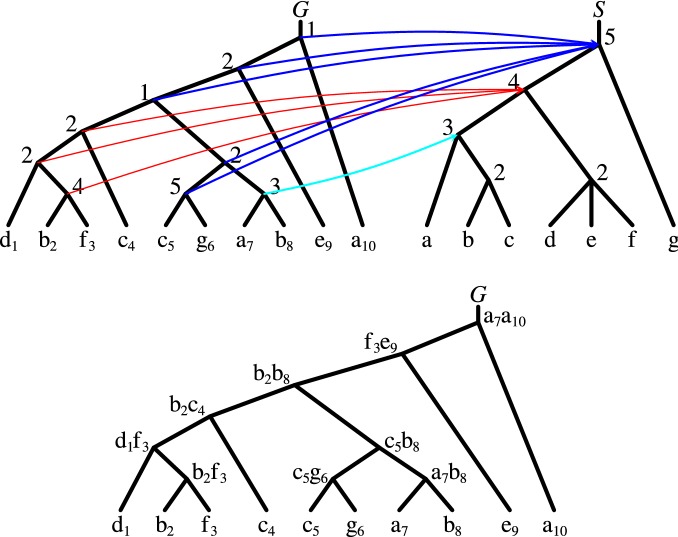
An example of a gene tree *G* and a species tree *S*. Top: The lca-mapping *M*. Each internal node of *S* is decorated with its rank based on the height of the corresponding subtree. Each internal node of *G* is decorated with the value of mapping *P*. Note that the parent of taxa *d*, *e* and *f* in *S* is a polytomy. Bottom: *G* in which each internal node is decorated with a pair of gene leaves that induces the value of mapping *P* in Algorithm 1 (line 7). For example, for the left child of the root, say *x*, $f_3e_9$ yields $P(x)=R(\mathop {\mathrm {lca}}_S(f,e))=2$

A node with more than two children is called a *polytomy*. For a polytomy *s* in a tree *S*, let *H*(*s*) be the set of all possible binary trees whose leaves are the children of *s*. For instance, if *s* is the polytomy node present in *S* from Fig. 2, then $H(s)=\{ (d,(e,f)), (e,(d,f)), (f,(d,e)) \}$. Let $H^*(S)$ be the set of all possible binary trees obtained from *S* by replacing each polytomy *s* with a tree from *H*(*s*). An element of $H^*(S)$ is called a *binarization* of *S*.

### Locus gain problems

In this section we introduce the parsimony framework for the (conditional) locus tree inference problem and a dynamic programming formula for solving the problem. We say that a gene tree *G* is *embeddable* (respectively, *conditionaly embeddable*) into a species tree *S*, if each node of *G* is a speciation (respectively, a speciation in some binarization of *S*). For instance, (*a*, (*b*, *c*)) is embeddable into (*a*, (*b*, *c*, *e*), *f*), while (*a*, (*a*, *b*)) is not. Since the polytomies in *S* can be resolved independently, we get the following result:

#### **Lemma 1**

*G is conditionally embeddable into S if and only if there is a binarization* $S'$ *of S such that G is embeddable into* $S'.$

#### *Proof*

($\Leftarrow$) Obvious. ($\Rightarrow$) First, observe that for any two nodes $g, g' \in G$, if *M*(*g*) and $M(g')$ are incomparable, then binarizing any node under *M*(*g*) will not change the mapping of any node below $g'$. Thus, we can binarize disjoint parts of *S* separately. Now, consider a maximal node *g* which maps to a non-binary node in *S* (i.e. a node *g* such that for any $g' \succ g$, $M(g')$ is binary). Since the parent of *g* is a speciation, the mappings of *g* and ${{\mathrm{\mathsf {sibling}}}}(g)$ are incomparable in *S*. Since *G* is conditionally embeddable, there exists a binary tree $T \in H(M(g))$ such that *g* becomes a speciation after replacing *M*(*g*) with *T*. From the definition of a speciation node it follows that, after such replacement, the children of *g* map onto disjoint parts of *S*, and subsequent polytomies can be resolved independently to obtain the desired binarization. $\square$

Every internal node *g* of *G* induces a set of *loss* events defined as nodes of the species tree strictly between *M*(*g*) and $M(\mathsf {parent}(g))$, plus *M*(*g*) if *M*(*g*) is a polytomy. The above definition yields a notion of the *loss cost*, denoted by $\Lambda (G,S)$, and defined as the total number of loss events required to embed *G* into *S*.

A gene tree may not be embeddable into a species tree due to duplications or HGTs. Our goal is to decompose a gene tree into a set of embeddable subtrees in the most parsimonious way. We say that a forest *F* is a *decomposition* of *G*, if $\bigcup _{T \in F}L(T)=L(G)$, and for every $T \in F$, $G|_{L(T)}=T$, where, for $A \subseteq L(G), \,G|_A$ is a tree having *A* as the set of leaves and $\{\mathop {\mathrm {lca}}_G(a, b)| a, b \in A\}$ as the set of internal nodes with the ancestor relation inherited from *G*. Decompositions can be equivalently obtained by tree edit operations as follows. Given a gene tree *G*, $X \subseteq E_G$ is called a *set of cuts* if no two edges in *X* share their top nodes.

Let *X* be a set of cuts from *G*. We define $G^X$ to be the graph obtained from *G* by removing all cuts from *G*, contracting all nodes with one parent and one child, and then removing all roots having exactly one child.

#### **Lemma 2**

*For every set of cuts X from* $G, \,G^X$ *is a decomposition of G*.

#### *Proof*

Removing all cuts from *G* leads to a set of disjoint and connected subgraphs of *G*. As no two cuts share the top node, each subgraph contains at least one leaf from *G*, i.e., there is no subgraph composed entirely of *G*’s internal nodes. On the other hand, each leaf from *G* is in exactly one subgraph. Internal nodes of *G* can be divided into four disjoint classes: (I) nodes disjoint with any cut, (II) bottom nodes of a cut incident to exactly one cut, (III) top nodes of a cut incident to exactly one cut, and (IV) nodes incident to exactly two cuts. Contracting nodes with exactly one parent and one child [(the group (III)] and removing single-child roots (IV) do not affect the leaves and the nodes from the group (I). Therefore, after these operations, each subgraph becomes either a binary tree or a leaf. It follows that $G^X$ is a forest such that $\bigcup _{T \in G^X} L(T) = L(G)$. Since each tree in $G^X$ is obtained from a connected subgraph of *G*, it inherits the ancestral relation from *G*. Furthermore, note that a node with one parent and one child or a root with one child cannot be the last common ancestor of any set of leaves. It follows that for any $T \in G^X$ we have $T = G|_{L(T)}$. $\square$

In the context of *X* every node of *G* can be uniquely associated with a node from $G^X$ by a mapping $\sigma ^X$ that maps a node *g* to the first non-removed node below *g*, connected by non-cut edges. Formally, $\sigma ^X(g)=\sigma ^X(g_1)$ if the edge $\langle g,g_2 \rangle$ is a cut from *X* and $g_1$ is the sibling of $g_2$, and $\sigma ^X(g)=g$, otherwise. By $M^X :G \rightarrow S$ we denote the “locus-aware” lca-mapping, given by $M^X(g)=M'(\sigma ^X(g))$, where $M'$ is the lca-mapping between $T \in G^X$ and *S* such that $\sigma ^X(g) \in T$ (see Fig. 1).

Consider a set of cuts *X* in *G*. We say that *X* *detaches* $g \in G$, or is *g-detaching*, if $\sigma ^X(g)$ is the root of some tree from $G^X$. For example, in Fig. 1, the cuts from the bottom example detach the parent of leaf $a_1$ (as $\sigma ^X(\mathsf {parent}(a_1))=b_2$ is a root in $G^X$), while the cuts from the top one do not ($\sigma ^X(\mathsf {parent}(a_1))=a_1$ is below the root).

We say that a decomposition is *consistent* (respectively, *conditionally consistent*) with a species tree *S* if for every $T \in F, \ T$ is (respectively, conditionally) embeddable into *S*. From the definition of $\sigma ^X$ we have:

#### *Remark 3*

Let $G^X$ be a decomposition consistent with *S*. Then, a set of cuts *X* detaches $g \in G$ if and only if every tree in $G^X$ is either disjoint with or entirely contained in *G*(*g*).

Given a species tree *S* and a gene tree *G* we define a *locus tree* with respect to *S* as a pair (*G*, *X*), where *X* is a set of cuts such that the decomposition $G^X$ is consistent with *S*. Locus trees which induce the same decompositions are considered equivalent. A locus tree represents the evolutionary history of a set of genes, while a cut corresponds to the creation of a new locus by gene duplication or horizontal gene transfer. The decomposition induced by a set of cuts represents independent evolutionary histories of different loci.

The definition of a locus tree presented in this work is formal, and does not always agree with the biological intuition. In “Relationship between decompositions and locus trees” section we show a set of cuts which cannot be directly explained by duplications and transfers, and requires additional evolutionary events to explain the decomposition (so-called *non-admissible* events). The topic of non-admissible events together with an algorithm for detecting them is covered in detail in “Relationship between decompositions and locus trees” section.

From Lemma 2 it follows that for each set of cuts there is a unique decomposition induced by this set. Conversely, for every decomposition *F* of *G* there exists a set of cuts *X* such that $F=G^X$. Inferring such a set from a given decomposition is straightforward by a bottom-up traversal of the gene tree. Therefore, we can consider decompositions as equivalent to locus trees. From the computational point of view, it is more natural to seek for optimal decompositions rather than sets of cuts.

In some applications (see e.g. “Results” section), given a locus tree (*G*, *X*) we seek for the $\prec$-maximal nodes, called *source nodes*, obtained from *G* after removing *X*. The set of all source nodes uniquely corresponds to the elements of $G^X$. For example, in Fig. 1, the top locus tree has two sources: the root of *G* and $b_2$, while in the bottom tree, we have three sources: the root of *G*, $a_1$ and the parent of $b_3$.

Given a decomposition *F*, we define the *total loss cost* as $\Lambda (F,S) = \sum _{T \in F} \Lambda (T,S)$. We can now define the *Locus Tree Inference* problem (*LTI*) in the parsimony framework:

#### **Problem 4**

(*Locus Tree Inference, LTI*) Given a gene tree *G* and a species tree *S*. Find the decomposition $F^*$ of *G* consistent with *S* having the minimal weighted cost $\Delta (G,S)$ $=$ $\mathbf {GAIN}\cdot |F^*| + \mathbf {LOSS}\cdot \Lambda (F^*,S)$ in the set of all decompositions of *G* consistent with *S*, where $\mathbf {GAIN}\ge 0$ and $\mathbf {LOSS}\ge 0$ are the weights of locus gain and locus loss events, resp.

Decompositions which satisfy the above conditions are referred to as *optimal*. Note that, from the mathematical point of view, one of the weights in the above cost function could be set to 1, and the other adjusted accordingly.

In the same way, for conditional consistency, we define the *Conditional Locus Tree Inference* problem (*CLTI*). The problems are equivalent if the input species tree is binary. From the algorithmic point of view, LTI is similar to the reconciliation with DTL (duplication-transfer-loss) scenarios [14] with no duplications. A transfer event corresponds to the creation of a tree in a decomposition forest. Additionally, we do not count loss events at the root of a new tree.

Our algorithm for solving LTI, described below, consists of several functions of $g \in G$, $s \in S$ and $\iota \in \{0,1\}$ which denotes whether a set of cuts detaches *g*:

D1: $\delta (g,s,0)$ is the minimal partial cost contribution of *G*(*g*) in the set of all *g*-detaching sets *X* such that $M^X(g)=s$.
D2: $\delta (g,s,1)$ as above but for non-*g*-detaching sets of cuts.
D3: $\delta ^\triangle (g,s,\iota )$ is the minimal value of $\delta (g,s',\iota )$ for $s' \preceq s$.
D4: $\delta ^\uparrow (g,s,\iota )$ is the minimal partial cost contribution of *G*(*g*) in the set of all *g*-detaching sets of cuts *X* such that $M^X(g) \preceq s$. For $\iota =1$, the cost additionally includes all losses created by $\sigma ^X(g)$ and associated with every species node $s'$ satisfying $M^X(g) \prec s' \preceq s$.

Below we present the dynamic programming formulas (DP Algorithm) for solving LTI. Here, *c*(*v*) is the set of children of *v* ($\emptyset$ for leaves). By ${\mathbf{{1}}}$ we denote the indicator function, that is, ${\mathbf{{1}}}[p]$ is 1 if *p* is satisfied and 0 otherwise.

$$\begin{aligned} \delta (g,s,\iota )&= {} \left\{ \begin{array}{l l} 0 &{} \text {if { g} is a leaf and M(g)\,=\,s}, \\ \min \{ \alpha , \gamma \} &{} \text {if { g} is not a leaf},\\ +\infty & {} \text {otherwise}, \end{array} \right. \\ \alpha &= {} {\mathbf{{1}}}[c(s) \ge 3] \cdot \mathbf {LOSS}\cdot \iota + \min _{ s',s'' \in c(s) \text {\ and\ } s' \ne s''} \delta ^\uparrow (g',s',1)+\delta ^\uparrow (g'',s'',1), \\ \gamma &= {} \mathbf {GAIN}+\min ( \delta ^\triangle (g',M(g'),0)+\delta ^\uparrow (g'',s,\iota ), \delta ^\triangle (g'',M(g''),0)+\delta ^\uparrow (g',s,\iota )),\\ \delta ^\uparrow (g,s,\iota ) &= {} \left\{ \begin{array}{l l} \delta (g,s,\iota ) &{} \text {if { s} is a leaf}, \\ \min \{ \delta (g,s,\iota ), {\mathbf{{1}}}[|c(s)|>1]\cdot \mathbf {LOSS}\cdot \iota + \min _{x \in c(s)} \delta ^\uparrow (g,x, \iota )\} &{} \text {otherwise,} \end{array} \right. \\ \delta ^\triangle (g,s,\iota ) &= {} \min \{ \delta (g,s,\iota ),\min _{x \in c(s)} \delta ^\triangle (g,x,\iota ) \}. \end{aligned}$$

#### **Theorem 5**

(Solution to LTI) *For every G and S:* $\Delta (G,S)=\min _{s \in S} \delta ^\triangle ({{\mathrm{\mathsf {root}}}}(G),s,0))+\mathbf {GAIN}.$

#### *Proof*

The proof is by induction on the structure of *G* and *S*, where the properties D1–D4 of all $\delta$’s are proved. We omit technical details. $\square$

#### **Theorem 6**

*The optimal cost can be computed in O* *(|G||S|m) time and O(|G||S|) space, where m is the maximal degree of a node from S.*

#### *Proof*

*Time:* We show that all values of $\delta$ functions can be computed in *O*(*m*) time. This is straightforward for all values except $\alpha$, where computing $\min$ potentially requires $O(m^2)$ time. This can be done, however, in *O*(*m*) time, by finding for each node $g'$ of *G*, the two children of *s* with the minimal and the second minimal value of $\delta ^\uparrow$ and choosing the minimal pair one among all four variants. *Space:* Obvious. $\square$

CLTI can be solved by an algorithm similar to the one presented above. It requires an additional case in $\delta$ for resolving duplications. To model a proper binarization of a polytomy in *M*(*g*), both children of *g* have to be mapped into disjoint sets of children of *M*(*g*). Such solution requires extending all $\delta$’s by a set of species nodes allowed for the mappings. In consequence, this approach has an exponential time and space complexity. We do not know if there is a polynomial time algorithm for CLTI. However, when the locus gain weight ($\mathbf {GAIN}$) is much greater than the loss weight ($\mathbf {LOSS}$), an efficient heuristic can be constructed, based on a mapping introduced in the next section.

### Ranked trees and rank-based mappings

Usually, when comparing trees, mappings based on their topologies are used (e.g., the lca-mapping). However, some biological species trees contain additional useful structure: the *taxonomic ranks*, like species, genus, or family. A species tree with taxonomic ranks assigned is sometimes called a *taxonomy*. Several major ranks are common to almost all living organisms. In this section, we propose a mathematical formalization of ranks and two rank-based mappings, which are useful in duplication inference and CLTI.

A *ranked species tree* is a species tree *S* in which every node *s* of *S* has assigned a small positive integer called *rank*, denoted $R(s)$, such that, for every *s* and $s'$, if $s \prec s'$ then $R(s)<R(s')$. We assume that the rank of the root of is $d>0$ and every leaf has rank 1.

After assigning integer numbers to the taxonomic ranks, a taxonomy becomes a ranked species tree. However, the latter concept is broader, as any species tree can be converted to a ranked one, for example by assigning ranks equal to the node depths. The theory and algorithms described in the following sections apply to any ranked tree, regardless of the way in which the ranks were assigned.

Let *G* be a gene tree and *S* be a ranked species tree. For a rank *r* and a leaf *t* in *S*, the unique directed path in *S* consisting of all taxa comparable with *t* having the rank lower than *r* will be called *an (evolutionary) r-lineage* of *t*. Note that every 1-lineage is empty. We say that leaf-taxa *t* and $t'$ are *separated* by the rank *r* if for every *x* from the *r*-lineage of *t* and every *y* from the *r*-lineage of $t'$, *x* and *y* are incomparable. Observe that every pair of leaf-taxa is separated by the rank of 1. Moreover, if *r* separates *t* and $t'$ then every rank lower than *r* also separates *t* and $t'$. For example, in Fig. 2 leaf-taxa *a* and *c* are separated by ranks 1, 2 and 3, but not by rank 4.

Let *g* be an internal node in *G* with children $g_1$ and $g_2$. The *highest separating rank* mapping $P :V_G \rightarrow \{1,2,\dots ,d\}$ is defined as

$$\begin{aligned} P(g) & = \max \left\{ r :r \,\,\, \text {separates every pair of leaf-taxa}\right. \\& \qquad \quad \left. \text {from} \,\, tax(g_1) \times tax(g_2) \right\} . \end{aligned}$$

The *lowest common rank* mapping $I :V_G \rightarrow \{1,2,\dots ,d\}$ is defined as $I(g)=R(M(g))$. We now present some fundamental properties of both mappings. Simple proofs are omitted for brevity.

#### **Lemma 7**

*Let* $\rho (t,t')$ *be the highest rank that separates leaf-taxa t and* $t'$ *and let g be an internal node of G with two children* $g_1$* and* $g_2.$* Then,*

(A) *For every leaf-taxa t and* $t',$ $\rho (t,t')=R(\mathop {\mathrm {lca}}_S(t,t')).$
(B) $$P(g)=\min _{\langle t,t' \rangle \in Q} \rho (t,t').$$
(C) $$I(g)=\max _{\langle t,t' \rangle \in Q} R(\mathop {\mathrm {lca}}_S(t,t')).$$
(D) $$P(g)=\min _{\langle t,t' \rangle \in Q} R( \mathop {\mathrm {lca}}_S(t,t')).$$
(E) $P(g)=1$ *if and only if* $\mathop {\mathrm {tax}}(g_1) \cap \mathop {\mathrm {tax}}(g_2) \ne \emptyset,$

* where* $$Q=\{ \langle t,t' \rangle \in tax(g_1) \times tax(g_2) \}$$.

#### *Proof*

For the proof of (A), let $r=R(\mathop {\mathrm {lca}}_S(t,t'))$. Then, by the definition, for every element *v* of *r*-lineage for *t*, $R(v)<R(\mathop {\mathrm {lca}}_S(t,t'))$. Hence, $v \prec \mathop {\mathrm {lca}}_S(t,t')$. And the same holds for the *r*-lineage of $t'$. Thus, both *r*-lineages consists of incomparable nodes. We conclude that the rank *r* separates *t* and $t'$. Note, that for ranks $r'>r$, both $r'$-lineages contain $\mathop {\mathrm {lca}}_S(t,t')$. Therefore, *r* is the highest rank separating *t* and $t'$. This completes the proof of (A). (B) follows from the definition of mapping *P* and (A). The proof of (C) is similar to (B). Both (D) and (E) follow from (A) and (B). $\square$

Taxonomic ranks have been used earlier for HGT detection [15]. In this work, the authors decorated nodes of the gene tree with the rank of the lowest taxon shared by each descendant leaf, equivalent with the *I* mapping. A high difference between the rank of a node and the one of its parent was one of the premisses for HGT. To the best of our knowledge, no mapping equivalent with the highest separating rank has been proposed to date.

### Computing mappings

Given a species tree *S* and a gene tree *G*, to compute *I* we can use the classical algorithm for lca-queries, in which, after a linear-time preprocessing, computing lca-queries can be completed in constant time [16]. We conclude that *I* can be computed in $O(|G|+|S|)$ time.

A naïve algorithm for computing *P*, based on Lemma 7, requires $O(|G||S|^2)$ time. Here, we propose an $O(d|G|+|S|)$ time solution. For two distinct leaves $l_1$ and $l_2$ of *G*, we write $l_1<_{p}l_2$ if $l_1$ is visited earlier than $l_2$ in prefix traversal of *G*. For instance, in Fig. 2 the leaves are linearly ordered starting from the left, i.e., $d_1<_p b_2<_p f_3 <_p \dots$.

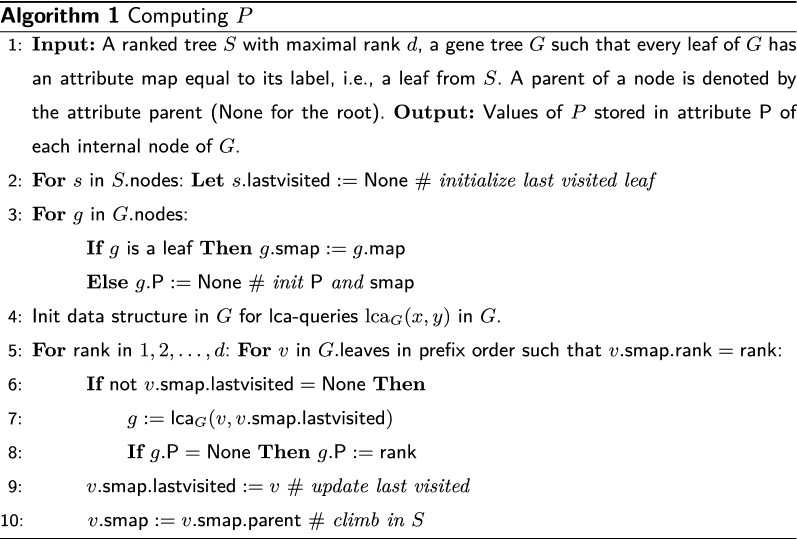



#### **Lemma 8**

*For a fixed* $s \in S$ *, the sequence of all assignment evaluations in line 9 such that* $v.\mathsf {smap}=s$ *induces a sequence of values v, denoted by* $v_1, v_2, \dots , v_k$ *such that: (I) the assignment* $s.\mathsf {lastvisited}:=v_i$ *is executed only when* $\mathsf {rank}=s.\mathsf {rank}$ *, (II)* $v_1<_p v_2<_p \dots <_p v_k$ *, and (III)* $\{v_1,v_2,\dots ,v_k\}=M^{-1}(L(s)).$

#### *Proof*

(I) is obvious by the condition in the second loop. By the condition in the inner loop, the order of leaves induced by a sequence of such assignments follows $<_p$. For every gene leaf *v*, $v.\mathsf {smap}$ is initially set to the label of *v*, i.e., *M*(*v*) (see line 3). Thus, if *s* is a leaf, i.e., $s.\mathsf {rank}=1$, then the assignment in line 9 sets the value of $v.\mathsf {lastvisited}$ if and only if the label of *v* is *s*. Thus for the leaves, (II) is satisfied. For (III), note that the line 10, ensures that every leaf *v* is assigned once to $s.\mathsf {lastvisited}$ of every node *s* of a species tree that is present on the path starting from *M*(*v*) and terminating in the root. Hence, $M^{-1}(L(s))$ $\subseteq$ $\{v_1,v_2,\dots ,v_k\}$. The other inclusion follows trivially from the fact that for a leaf *v*, $v.\mathsf {smap}$ is originally set to *M*(*v*) and *v* cannot be assigned to a node incomparable with *M*(*v*). $\square$

#### **Lemma 9**

*For every internal node g,* $P(g)=g.\mathsf {P}.$

#### *Proof*

Let $g'$ and $g''$ be the left and the right child of *g*, respectively. The proof is by induction on the rank $r=1,2,\dots ,d$, where *d* is the highest rank in *S*. Let $r=1$. Assume that $P(g)=1$, we show that $g.\mathsf {P}=1$. Let $s \in \mathop {\mathrm {tax}}(g') \cap \mathop {\mathrm {tax}}(g'')$. Then, by Lemma 8, let $\Lambda _s$ be the sequence $\{v_1,v_2,\dots ,v_k\}$ of all leaves assigned to $s.\mathsf {lastvisited}$ such that $M(v_i)=s$ and ordered by $<_p$. Clearly, the list has the leaves from both subtrees of *g*. Thus there is an index $j<k$, such that $v_j \in L(g')$ and $v_{j+1} \in L(g'')$. Thus $\mathop {\mathrm {lca}}_G(v_j,v_{j+1})=g$. Now, in line 8, when *v* is $v_{j+1}$ then $v.\mathsf {smap}.\mathsf {lastvisited}$ is $v_j$. In such a case, either $g.\mathsf {P}$ is $\mathsf {None}$ and $g.\mathsf {P}$ will be set to 1, or $g.\mathsf {P}$ is already set. However, it can be only 1. This completes the first part of the proof.

Assume that $P(g)=r$ and for every *q*, such that $P(q)<r$, we have $P(q)=q.P$. For every $v \in L(g')$ and $w \in L(g'')$, $R(\mathop {\mathrm {lca}}_S(M(v),M(w)))\ge r$. Thus $g.P=\mathsf {None}$, when Algorithm 1 starts the main loop with $\mathsf {rank}=r$. From Lemma 7, there is a pair taxa $\langle t_1,t_2 \rangle \in \hat{g}$ such that $s=\mathop {\mathrm {lca}}_S(t,t')$ and $R(s)=r$. Thus, there are two leaves $a_1$ and $a_2$ in *G* such that for each *i*, $M(a_i)=t_i$ and $\mathop {\mathrm {lca}}(a_1,a_2)=g$, i.e. $a_1 \preceq g'$ and $a_2 \preceq g''$. Similarly, to the first step, the leaves from $M^{-1}(L(s))$ are all visited and set to $s.\mathsf {lastvisited}$ according to the order $<_p$. The sequence contains elements $a_1$ and $a_2$, therefore again there is *j* separating leaves from both subtrees of *g*. The rest of the proof is analogous: in line 8 either *g*.*P* is already set to *r* (if there was another $s'$, processed before *s*, with $R(s')=r$ satisfying the same properties as *s*) or it will be set to *r*. This completes the proof. $\square$

#### **Lemma 10**

*Algorithm 1 requires* $O(d|G|+|S|)$ *time and* $O(|G|+|S|)$ *space.*

#### *Proof*

*Time:* Lines 1–4 have $O(|G|+|S|)$ time complexity, while the body of the inner loop needs *O*(1) time. *Space:* Algorithm 1 uses only a few node attributes plus the lca-query data structure of the size *O*(|*G*|). $\square$

### Classification of gene duplications

Several methods for reconciliation with non-binary gene trees have been proposed [17–22]. However, reconciliation with non-binary species trees is harder to model. This is because a polytomy may represent a lack of knowledge about the order of speciations, and therefore some duplication nodes may correspond to biological speciations. This motivates a further classification of duplication nodes into conditional and required duplications [10]. In our model, we assume that the species tree is ranked. This approach can be applied to any species tree by assigning ranks, e.g. based on the node depth.

When reconciling a gene tree *G* with every binarization of *S*, if *g* from *G* is a duplication in every reconciliation, then *g* is called a *required duplication*. Similarly, if *g* is a duplication in at least one but not all reconciliations, we say that *g* is a *conditional duplication*. Note that *G* is conditionally embeddable in *S* if and only if each node in *G* is either a speciation or a conditional duplication.

In this section, we show how *P* and *I* can be used to solve the problem of gene duplication classification when the species tree has possible polytomies.

#### **Lemma 11**

*For an internal node g from a gene tree G, the following conditions are equivalent: (A1)* $P(g)=I(g)$*, (A2) every subtree rooted below M(g) contains taxa from at most one child of* *g*, i.e., for every $s \prec M(g)$*, if* $L(s) \cap \mathop {\mathrm {tax}}(g_1) \ne \emptyset$ *then* $L(s) \cap \mathop {\mathrm {tax}}(g_2)=\emptyset$*, where* $g_1$ *and* $g_2$ *are the children of g, and (A3) for every* $\langle t,t' \rangle \in tax(g_1) \times tax(g_2),$ $\mathop {\mathrm {lca}}_S(t,t')=M(g)$.

#### *Proof*

*(A1)* $\Rightarrow$ (A2). Assume that $s \prec M(g)$ and there are two leaves *t* and $t'$ in *L*(*s*) such that $t \in \mathop {\mathrm {tax}}(g_1)$ and $t' \in \mathop {\mathrm {tax}}(g_2)$. Hence, $\langle t,t' \rangle \in tax(g_1) \times tax(g_2)$ and $\mathop {\mathrm {lca}}_S(t,t') \preceq s \prec M(g)$. Thus, $P(g)<I(g)$, a contradiction. *(A2)* $\Rightarrow$ (A3). Let $\langle t,t' \rangle \in \hat{g}$. Then, *t* and $t'$ are leaves from two different subtrees rooted below *M*(*g*). Therefore, $\mathop {\mathrm {lca}}_S(t,t')=M(g)$. *(A3)* $\Rightarrow$ (A1). It follows immediately from Lemma 7. $\square$

Note that the above Lemma also holds when $P(g)=I(g)=1$, i.e. when an internal node *g* is mapped to a leaf of *S*. In such a case the condition (A2) is satisfied trivially.

#### **Lemma 12**

*Let g be an internal node of G such that* $P(g)=I(g)$*. Then, g is a speciation if and only if M(g) is an internal node and there are* *exactly two subtrees rooted at children of M(g) having nodes from* $\mathop {\mathrm {tax}}(g).$

#### *Proof*

$(\Rightarrow )$. We have that *g* is an internal node. In such a case $I(g)>1$ and *M*(*g*) is an internal node. Then, by (A2) from Lemma 11, every child of *M*(*g*) has taxa present in at most one child of *g*. There are at least two children of *M*(*g*) satisfying this property. If there are more than two, then one child of *g*, say $g_1$, has taxa from at least two children of *M*(*g*). Hence, $M(g_1)=M(g)$ and *g* is a duplication node, a contradiction. $(\Leftarrow )$. Similarly, if *M*(*g*) is an internal node, then by (A2), the mappings of the children of *g* are incomparable and located below *M*(*g*). Therefore *g* cannot be a duplication. $\square$

We have a symmetric property whose proof is similar to the previous one.

#### **Lemma 13**

*Let g* *be an internal node of G such that* $P(g)=I(g)$ *. Then, g is a duplication node if and only if either M(g) is a leaf or* *M(g) is an internal node and there are at least three subtrees rooted at a child of* *M(g) having nodes from* $\mathop {\mathrm {tax}}(g).$

Finally, we have the main property.

#### **Theorem 14**

(Classification Theorem) *Let g be an internal node of* *G. Then, (C1) If* $P(g)=I(g)=1$ *or*$P(g)<I(g)$ *then g is a required duplication. (C2) If* $P(g)=I(g)>1$*, then g is a duplication if and only if g is a conditional duplication.*

#### *Proof*

*(C1)* If $P(g)=I(g)=1$, then *g* is mapped to a leaf. Hence, every leaf below *g* has the same label. Thus, in every binarization of *S*, *g* is a duplication. Assume that $P(g)<I(g)$. Then *M*(*g*) is an internal node in *S*, having at least three taxa in *L*(*M*(*g*)) (otherwise, the two children of *M*(*g*) are leaves and $P(g)=I(g)=2$). We can assume that there are three leaves $t,t' \in \mathop {\mathrm {tax}}(g_1)$ and $t'' \in \mathop {\mathrm {tax}}(g_2)$ such that $\mathop {\mathrm {lca}}_S(t',t'') \prec \mathop {\mathrm {lca}}_S(t,t',t'')$. This property holds for every binarization *T* of *S*, where the possible polytomy *M*(*g*) is resolved. Moreover, in every *T*, $M(g_1) \succeq \mathop {\mathrm {lca}}_T(t,t',t'')$, thus $M(g_1)$ is comparable with $M(g_2) \succeq t''$. Thus, $M(g)=\max (M(g_1),M(g_2))$ and *g* is a duplication node.

*(C2*,$\Leftarrow$). If *g* is a conditional duplication, then it is a duplication by definition. *(C2,*$\Rightarrow$). Assume that *g* is a duplication, then by condition (A2) from Lemma 11, the children of *M*(*g*) can be clustered into three disjoint sets *X*, $X'$ and $X''$ such that every node from *X* has no taxa present in $\mathop {\mathrm {tax}}(g)$, every node of $X'$ has taxa from $\mathop {\mathrm {tax}}(g')$ but not from $\mathop {\mathrm {tax}}(g'')$ and analogously every node of $X''$ has taxa from $\mathop {\mathrm {tax}}(g'')$ but not from $\mathop {\mathrm {tax}}(g')$, where $g'$ and $g''$ are the children of *g*. Also, by Lemma 13 at least one among $X'$ and $X''$, say $X'$, has at least two elements. Consider a binary tree *T* in *H*(*M*(*g*)), such that all elements of $X'$ and $X''$ are located on the left and the right subtree of *T*, respectively. Then, $\mathop {\mathrm {lca}}_T(X')$ and $\mathop {\mathrm {lca}}_T(X'')$ are incomparable. Thus, in such a binarization of *S*, $g'$ and $g''$ maps below *M*(*g*), and *g* is a speciation node. Similarly, it can be shown that there exists a tree in *H*(*M*(*g*)) in which *g* is a duplication. $\square$

Based on Algorithm 1, classification theorem leads to a natural $O(d|G|+|S|)$ time solution for the inference of required and conditional duplications when reconciling a given binary gene tree with a species tree. This improves the known $O(|G|(d+m)+|S|)$ time algorithm from [10], where *m* is the maximal degree of a node from *S*. The improvement is beneficial for highly polytomic species trees. For example, as of 04.28.2017, the genus *Aspergillus* has 1950 children species in the NCBI Taxonomy.

### Heuristic for CLTI

In this section, we propose the heuristic algorithm for CLTI when the locus gain weight is much higher than the loss weight. The algorithm is based on the following lemma, which follows directly from Theorem 14:

#### **Lemma 15**

*Tree G is conditionally embeddable in S if and only if, for all internal nodes g in G,* $I(g)=P(g)>1.$

Algorithm 2 is a greedy approach that iteratively finds the minimal nodes *g* such that $P(g) < I(g)$ or $I(g) = 1$ and detaches an embeddable subtree below each node. Note the following:

#### *Remark 16*

Let *G* be conditionally embeddable in *S*. Let $\hat{\Lambda }(G, S) = |L(M({{\mathrm{\mathsf {root}}}}(G)))| - |L(G)|$. Then, $\Lambda (G, S) \le \hat{\Lambda }(G, S)$.

Let $T_1\setminus T_2$ denote tree $T_1$ with detached subtree $T_2$. Then, $\hat{\Lambda }(G', S) + \hat{\Lambda }(G(g)\setminus G', S)$ is an estimate of the partial loss cost induced by detaching subtree $G'$. The detached subtree in Algorithm 2 is chosen to minimize this estimate. To limit the complexity of a single step, we consider only subtrees rooted at vertices at a close neighborhood of *g*.

#### **Lemma 17**

*Algorithm 2 returns a decomposition conditionally consistent with S in* $O(ad|G| + a|S|)$ *time, where a is the number of recomputations of I and P mappings.*

#### *Proof*

*Correctness* It follows immediately from the fact that each *G*(*w*) detached in the inner loop is conditionally embeddable. *Time* Let *g* be an element of *Z*. Then, as *G* is binary, there are at most six edges $\langle v,w \rangle$ adjacent to a child of *g*. (Case I) If $v=g$ and *w* is the child of *g*, then both trees *G*(*w*) and $G(g)\setminus G(w)$ are conditionally embeddable by the assumption that *g* is the minimal node such that *G*(*g*) is not conditionally embeddable. This completes the proof for $v=g$. (Case II) For the remaining case, there at most four grandchild edges connecting a child *v* with a grandchild *w* of *g*. We can arbitrarily index them by 1,2,3 and 4. Again, *G*(*w*) is conditionally embeddable, however, it is unclear for $T=G(g)\setminus G(w)$. By Lemma 15, it is sufficient to check whether, for the root *t* of *T*, $I_T(t)=P_T(t)>1$, where the mappings are from $V_T$. $I_T(t)$ can be computed in *O*(1) time by the rank of $\mathop {\mathrm {lca}}_S(M(v.{{\mathrm{\mathsf {sibling}}}}),M(w.{{\mathrm{\mathsf {sibling}}}}))$, however, for $P_T :V_T \rightarrow \{1,2,\dots ,d\}$ we need to use Algorithm 1. To avoid quadratic time, consider the following additional steps after line 4.

1. For each $i \in \{1, 2, 3, 4\}$, create a copy $G_i$ of *G*.
2. For each *g* in *Z*, remove the *i*-th grandchild edge $\langle v,w \rangle$ and *G*(*w*) from $G_i$.
3. For every *i*, let $P_i$ be the mapping *P*, denoted $P_i$, computed by Algorithm 1.

Then, when the *i*th grandchild edge $\langle v,w \rangle$ is processed in the inner loop, the value $P_T(t)$ is $P_i(t)$. We conclude that each step of the main loop (lines 2–6), requires 4 additional runs of Algorithm 1. $\square$

A significant advantage of Algorithm 2 is the space complexity, which is $O(|G| + |S|)$. This makes the heuristic suitable for trees with hundreds or thousands of nodes. Note that in Lemma 17, *a* is pessimistically equal to the height of the gene tree, which makes this algorithm asymptotically quadratic. However, in applications, *a* is expected to be a small integer. This expectation is valid e.g. in cases where evolutionary events occur less frequently than speciations. Note, however, that it may not be valid for genes which exhibit particularly complex evolutionary history, like transposons.

### Relationship between decompositions and locus trees

An evolutionary scenario is the set of evolutionary events which have shaped the observed gene tree. Usually, a scenario is represented by assigning duplication labels to some nodes of the gene tree, and transfer labels to some of its edges.

**Fig. 3:**
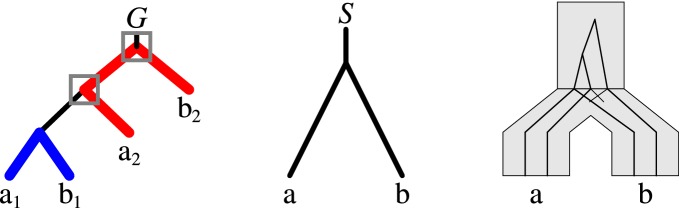
An example of a decomposition of a gene tree *G* into two trees $(a_1,b_1)$ and $(a_2,b_2)$ for which evolutionary scenarios require one additional duplication located on the internal node of the decomposition forest (here the root of *G*). The scenario with the minimal number of duplications (here 2) is depicted on the left in the form of the embedding of *G* into *S* [12]

Each evolutionary scenario, understood as a set of duplication nodes and transfer edges, induces a decomposition of the gene tree into a set of trees representing evolutionary histories of different loci. A set of cuts for such decomposition consists of all transfer edges and one edge below each duplication. One could reasonably expect that a converse relation holds, i.e. that each decomposition induces an evolutionary scenario and a similar set of cuts. This, however, turns out to not be true. The decomposition depicted in Fig. 3 requires adding additional duplication nodes with no cuts below them. Thus, one of the trees in this decomposition corresponds to the evolutionary history of two loci. In this section, we give preliminary results on the relationship between decompositions and locus trees. We begin with formalizing the notion of an evolutionary scenario. Next, we formally define the non-admissible events induced by a decomposition, and show and algorithm of detecting them. The number of non-admissible events measures how well a decomposition agrees with biological intuition behind evolutionary scenarios.

The formal definition of a DTL scenario presented below is adopted from [23, 24] with the difference that we focus more on the event based conditions.

#### **Definition 18**

(*DTL scenario, from* [25]) A DTL scenario, or scenario, for a binary tree *G*, and a tree *S* and a labelling ${\mathcal L} :L_G \rightarrow L_S$ is a tuple $\langle {{\mathrm{\mathrm {M}}}},\Sigma ,\Delta ,\Theta ,\xi \rangle$ such that ${\mathcal L} :L_G \rightarrow L_S$ is the leaf labelling function, ${{\mathrm{\mathrm {M}}}}:V_G \rightarrow V_S$ is a mapping that extends ${\mathcal L}$, $\{\Sigma ,\Delta ,\Theta \}$ is a partition of $I_G$ into speciation, duplication and transfer nodes, respectively, and $\xi :\Theta \rightarrow V_G$ determines the termination node of a transfer in *G*, subject the following conditions. For any $g \in I_G$ such that $c_1$ and $c_2$ are the children of *g*, let $s=\mathop {\mathrm {lca}}_S({{\mathrm{\mathrm {M}}}}(c_1),{{\mathrm{\mathrm {M}}}}(c_2))$. Then,

- We have $g \in \Sigma$ if and only if the mappings of the children of *g* are incomparable, and $s={{\mathrm{\mathrm {M}}}}(g)$.
- If $$g \in \Delta$$ then $$s \preceq {{\mathrm{\mathrm {M}}}}(g)$$.
- If $g \in \Theta$ then $\xi (g)$ is a child of *g*, ${{\mathrm{\mathrm {M}}}}({{\mathrm{\mathsf {sibling}}}}(\xi (g))) \preceq {{\mathrm{\mathrm {M}}}}(g)$, and ${{\mathrm{\mathrm {M}}}}(g)$ and ${{\mathrm{\mathrm {M}}}}(\xi (g))$ are incomparable. The edge $\langle g,\xi (g) \rangle \in E_G$ is called a transfer edge.

**Fig. 4:**
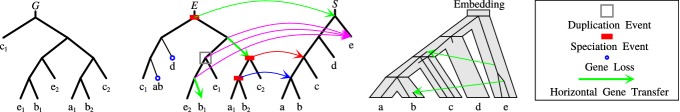
An example of a DTL scenario *E* for a gene tree *G* and a species tree *S*. *E* has two HGTs, one duplication, and three speciation events. The scenario is shown with the mapping *M* depicted for the internal nodes only. While the gene loss events are not formally modeled here, they are required when embedding a gene tree into a species tree according to a given DTL scenario. Therefore, our visualizations of DTL scenarios are extended by the required loss events (see also the scenario $E_3$ in Fig. 5)

The above conditions denote the speciation, duplication and horizontal gene transfers events, respectively. An example of an evolutionary scenario has been depicted in Fig. 4. In DTL scenarios, a *vertical* descent is modeled by the condition that the mapping of a child is below or equal to the mapping of its parent. The condition holds for the children of speciation and duplication nodes. A destination of a transfer node *g* is defined by the function $\xi$. Therefore, we require that both the mapping of *g* and the mapping of $\xi (g)$ are incomparable. Note that, the above definition of a DTL scenario does not exclude cyclic HGT scenarios, i.e. scenarios with at least two conflicting transfer edges. Conflicting edges occur e.g. when the acceptor of the first transfer is above the donor of the second one, and the acceptor of the second transfer is above the donor of the first one. Such scenarios are impossible to occur in nature, because the acceptors and donors need to be present at the same time point. If the optimal cost is defined as the minimal weighted sum of numbers of HGT and duplication events, then, for a given gene tree and a species tree, it can be computed in *O*(|*G*||*S*|) time [23, 24], while the problem for acyclic scenarios is NP-hard [24].

### Validation of decomposition

Let *F* be a decomposition of a gene tree *G*. Then, by the definition of decomposition every node of *F* is a node of *G*. Such nodes will be called *F-internal*.

Now, we can determine how a given decomposition is evolutionarily congruent by searching for the DTL scenarios that preserve the largest number of speciation events in the forest and the minimal number of duplication and HGT events outside of it. Given a scenario *E* for *G* and *S*, we say that an event $g \in I_G$ is *admissible* if

- $g \in \Delta$ and *g* is not *F*-internal.
- $$g \in \Theta$$ and $$\langle g,\xi (g) \rangle \notin E_F$$.
- $g \in \Sigma$ and *g* is *F*-internal.

Given that $|\Sigma |+|\Delta |+|\Theta |=|G|-1$, the decomposition congruency can be equivalently expressed in terms of minimizing the number of non-admissible events.

#### **Problem 19**

(*Validation of decomposition*) Given a gene tree *G*, a species tree *S* and a decomposition *F* of *G* consistent with *S*. Find a DTL scenario for *G* and *S* that minimizes the number of non-admissible events.

**Fig. 5:**
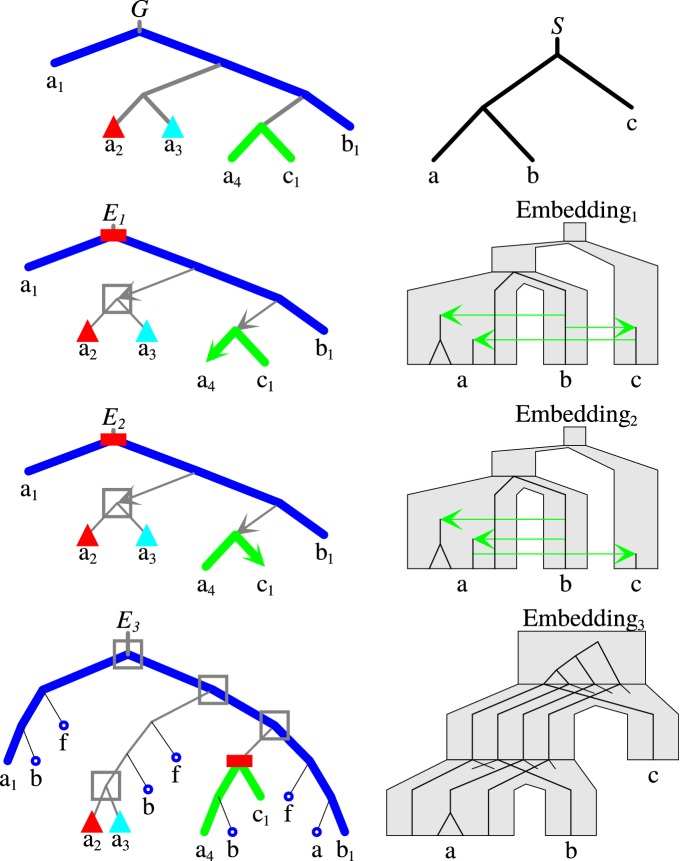
An example of the validation of a decomposition with one non-admissible event. *Top* A species tree *S* and a gene tree *G* decomposed into 4 trees $(a_1,b_1)$ (blue), $(a_4,c_1)$ (green), $a_2$ (red) and $a_3$ (light blue). *Bottom (3 rows)* DTL scenarios $E_1$-$E_3$ with embeddings. In scenarios $E_1$ and $E_2$ there is one non-admissible HGT terminating in $a_4$ and $c_1$, respectively, while $E_3$ has one non-admissible duplication at the root

Examples of such scenarios are depicted in Fig. 5. The above problem can be solved by a dynamic programming algorithm in *O*(|*G*||*S*|) time similar to the decomposition problem and the reconstruction of DTL scenarios [23, 25]. Similarly to the decomposition formulas for $g \in G$ and $s \in S$ we have the following properties of the $\sigma$’s:

V1: $\sigma ^\triangle (g,s)$ is the minimal number of non-admissible events located in *G*(*g*) for the scenarios between *G*(*g*) and *S*(*s*).
V2: $\delta (g,s)$ as above but under assumption that *g* is mapped to *s*.
V3: $\delta ^\rightarrow (g,s)$ is the minimal number of non-admissible events located in *G*(*g*) in the set of all scenarios for *G*(*g*) and *S*(*x*) where *x* is incomparable with *s*.

Below we present the dynamic programming formulas for the validation of decompositions. Here $g'$ and $g''$ are the children of *g*, $\alpha$ represents the case when *g* is a speciation, $\beta$—a duplication and $\gamma$—an HGT.

$$\begin{aligned} \sigma (g,s)&= {} \left\{ \begin{array}{l l} 0 &{}\quad \text {if} \ g \ \text{is a leaf and} \ M(g) =s, \\ \min \{ \alpha , \beta , \gamma \} &{}\quad \text {if} \ g \ \text{is not a leaf},\\ +\ \infty &{}\quad \text {otherwise}, \end{array}\right. \\ \text {where}&\\ \alpha &= {} {\mathbf{{1}}}[g \text {\ is not} \ F{\text{-internal}}] + \min _{ s',s'' \in c(s), s'\ne s''} \sigma ^\triangle (g',s')+\sigma ^\triangle (g'',s''), \\ \beta &= {} {\mathbf{{1}}}[g \text {\ is} \ F{\text{-internal}}] + \min _{ s' \in c(s) \cup \{s\} } \sigma ^\triangle (g',s')+\sigma (g'',s), \\ \gamma &= {} \min {\mathbf{{1}}}[\langle g,g' \rangle \in E_F]+\sigma ^\rightarrow (g',s)+\sigma ^\triangle (g'',s), {\mathbf{{1}}}[\langle g,g'' \rangle \in E_F]+\sigma ^\rightarrow (g'',s)+\sigma ^\triangle (g',s) \}, \\ \sigma ^\triangle (g,s) &= {} \min _{x \preceq s} \sigma (g,x), \\ \sigma ^\rightarrow (g,s) &= {} \min _{x \text {\ and} \ s \ {\text{are incomparable}}} \sigma (g,x). \end{aligned}$$

#### **Theorem 20**

*Given a gene tree G, a species tree S and a decomposition F of G consistent with S. The minimal number of non-admissible events equals* $\min _{s \in S} \sigma ^\triangle ({{\mathrm{\mathsf {root}}}}(G),s)).$

#### *Proof*

The proof is by induction on the structure of *G* and *S*, where the properties V1–V3 of all $\sigma$’s are proved. We omit technical details. $\square$

#### **Theorem 21**

*The number of non-admissible events can be computed inO* *(|G* *||S* *|m) time and* *O* *(|G* *||S* *|) space, where m is the maximal degree of a node from* *S.*

#### *Proof*

See the proof of Theorem 6. $\square$

## Results

We have run numerical simulations to assess the performance of the proposed algorithms. First, we have run the heuristic and dynamic programming algorithms on pairs of random trees to compare the inferred forest sizes and loss costs. Next, we have performed simulations of realistic evolutionary scenarios to check the correctness of the algorithms’ results.

The optimal decomposition is seldom unique. Therefore, when analyzing the dynamic programming algorithm, for each gene tree-species tree pair we picked one of the optimal decompositions randomly. The heuristic algorithm always returns a single decomposition.

### Comparison of algorithms

In the case of binary species trees, conditional embeddability is identical to strict embeddability, and both locus tree inference algorithms can be compared experimentally.

**Fig. 6:**
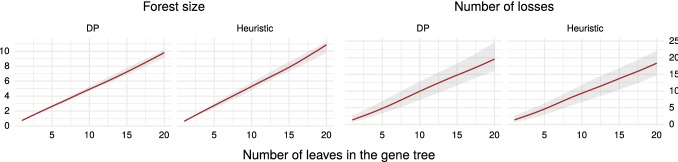
Comparison of DP and heuristic algorithms for binary species trees in terms of forest size |*F*| and numbers of losses $\Lambda (F, S)$. The brown line depicts the median cost; the grey ribbon depicts the 90% confidence interval. The weights in the DP algorithm have been set to $\mathbf {GAIN}=1000, \mathbf {LOSS}=1$. The plots have been smoothed with cubic splines

For each $|L(G)|=1,\dots ,20$ and $|L(S)|=10$ we have generated 100 pairs of random trees under the Yule-Harding model. The leaves of *G* have been assigned to leaves of *S* randomly. The numbers of losses for heuristic algorithm have been computed using a modification of the DP algorithm for LTI. The inferred costs are shown in Fig. 6.

The costs are similar for both algorithms. The forest size is approximately half the number of leaves in *G*. Using linear regression, we have determined that, on average, the inferred forest size is equal to 0.47|*L*(*G*)| for DP and 0.53|*L*(*G*)| for the heuristic. Large forest sizes in these examples can be explained by the fact that both gene and species trees are simulated independently, and most trees in the forests contain only one or two leaves.

The number of losses is slightly smaller for the heuristic algorithm (on average 0.98|*L*(*G*)| for DP and 0.90|*L*(*G*)| for the heuristic). We hypothesize that the reason for this is that the greedy approach tends to detach more concise trees.

### Detecting evolutionary events

To validate our approach under more realistic conditions, we have run simulations of evolutionary scenarios using the GenPhyloData tool [26]. The tool simulates species trees under a stochastic birth-and-death model, in which each leaf gets duplicated or lost with a constant rate. The total rate of duplication (loss) is equal to the duplication (loss) rate times the number of leaves at a given time point (see [27] for details). When the loss rate is equal to zero, this process reduces to the Yule-Harding model. In the case of the species tree, a branch duplication models a speciation, and branch loss models an extinction.

In each branch of the species tree, a similar birth-and-death process models the gene duplication and loss events. An additional Poisson process models the occurrence of horizontal gene transfer events. A recipient of the transferred gene is picked uniformly from species alive at a given time point.

**Fig. 7:**
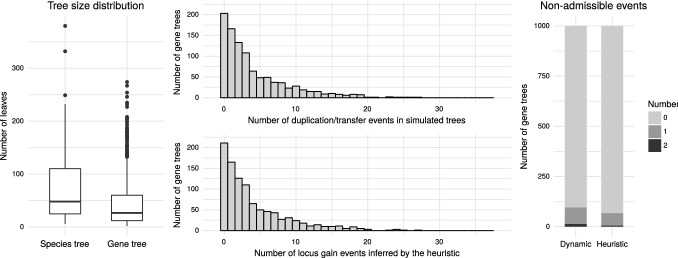
Results of the simulations of evolutionary scenarios. Left: Distribution of numbers of leaves in simulated gene and species trees. Middle top: Numbers of duplication or transfer events in the simulated evolutionary scenarios. Middle bottom: Numbers of locus acquisition events (i.e., forest size minus one) inferred by the heuristic algorithm. Right: Numbers of non-admissible events induced by decompositions inferred by the dynamic programming and the heuristic algorithm

Using GenPhyloData, we have simulated 100 species trees under a birth-and-death model with the birth rate 5 and the death rate 1. Trees have been pruned to remove extinct lineages. This procedure resulted in a set of binary species trees with approximately 75 leaves on average (see Fig. 7).

For each species tree, we have simulated 10 gene trees with duplication rate 0.4, transfer rate 0.1 and loss rate 1.2. Trees have been pruned to remove lost genes. We have obtained a set of 1000 binary gene trees with approximately 43 leaves and 4 duplication or transfer events on average (see Fig. 7). The maximum number of events in a single gene tree was 27.

The simulated gene trees have been decomposed with respect to the corresponding species trees using our heuristic and dynamic programming algorithms. In both cases, the numbers of duplication/transfer events were similar to the inferred number of new loci (i.e. forest size minus one, accounting for the “base” locus at the root of the gene tree). The forest sizes inferred by the heuristic algorithm have been depicted in Fig. 7. The number of locus acquisition events has been inferred properly in 82.6 and $82.7\%$ of the gene trees by the heuristic and dynamic programming algorithm, respectively, and differed by at most one event in 98.3 and $98.2\%$ cases. The dynamic programming algorithm underestimated the number of evolutionary events slightly more often than the heuristic one. The number of inferred events was lower than the actual number in 10.7 and $11.9\%$ of trees for the heuristic and dynamic programming algorithms, respectively.

To further validate our results, we have checked whether the source nodes inferred by the decomposition algorithms correspond to simulated evolutionary events. Note that, from the definition, two source nodes cannot be siblings (see “Locus gain problems” section for the definition of a source node). A properly predicted source node is either the end of a transfer edge, or a child of a duplication node. We say that a properly predicted source node is a *true positive* event. Consequently, an event that failed to be predicted is a *false negative*; a speciation that is classified as a source is a *false positive*; and a properly predicted speciation is a *true negative*.

Note that for a transfer edge, a decomposition algorithm might classify the sibling of the edge’s end as a source node. In this case, we assume that the algorithm is wrong twice: first, it incorrectly classifies a node as a source (a false positive), and second, it fails to detect an evolutionary event (a false negative). To account for this assumption, we have adopted the following convention. A duplication event corresponds to:

1. One true positive and one true negative event if one of its children is a source node,
2. One true negative and one false negative if none of its children are source nodes.

A transfer event corresponds to:

1. One true positive and one true negative if one of its children is a source node, and this child is the end of the transfer edge,
2. One false positive and one false negative if one of its children is a source node, and the end of the transfer edge is the sibling of the source,
3. One true negative and one false negative if none of its children are source nodes.

The results for both algorithms have been summarized in Table 1. In the case of the heuristic algorithm, $93.2\%$ of the duplication/transfer nodes were correctly detected. The positive predictive value of the heuristic approach (proportion of inferred locus gain events corresponding to true duplication/transfer events) was equal to $94.4\%$. In the case of the dynamic programming algorithm $86.7\%$ of duplication/transfer nodes were correctly classified as corresponding to a new locus, and the positive predictive value was equal to $88.4\%$.

**Table 1:** The confusion table of gene tree nodes classification

	Event	Speciation	Sum
DP			
Locus	3584	471	4055
Speciation	552	78,591	79,143
Sum	4136	79,062	83,198
Heuristic			
Locus	3858	229	4087
Speciation	278	78,833	79,111
Sum	4136	79,062	83,198

Event/locus: duplication or transfer nodes corresponding to new loci (true positive); event/speciation: duplication or transfer nodes not corresponding to new loci (false negative); speciation/locus: speciation nodes corresponding to new loci (false positive); speciation/speciation: speciation nodes not corresponding to new loci (true negative)

We have further verified the correctness of our approach by investigating the numbers of non-admissible events induced by the decompositions. The results are depicted in Fig. 7. Overall, there was no non-admissible event in $93.2\%$ of decompositions returned by the heuristic algorithm and $90.4\%$ of decompositions returned by the dynamic programming one.

A likely reason for the worse performance of the dynamic programming algorithm is the random choice of optimal decomposition. The number of non-admissible events might in future be used as an additional criterion for the optimality of the decomposition.

### Applications of evolutionary history decomposition

We have compared our approach with a state-of-the-art reconciliation program, Notung 2.9 [28]. We have analyzed the evolution of the gene family of an aminotransferase from a fungus *Penicillium lilacinoechinulatum* [GenBank:ABV48733.1] with a history marked by HGT events [9, 29]. Homologs of the protein sequence have been found using BLASTp suite. For analysis, we have chosen 45 closest homologs from 20 fungal species.

**Fig. 8:**
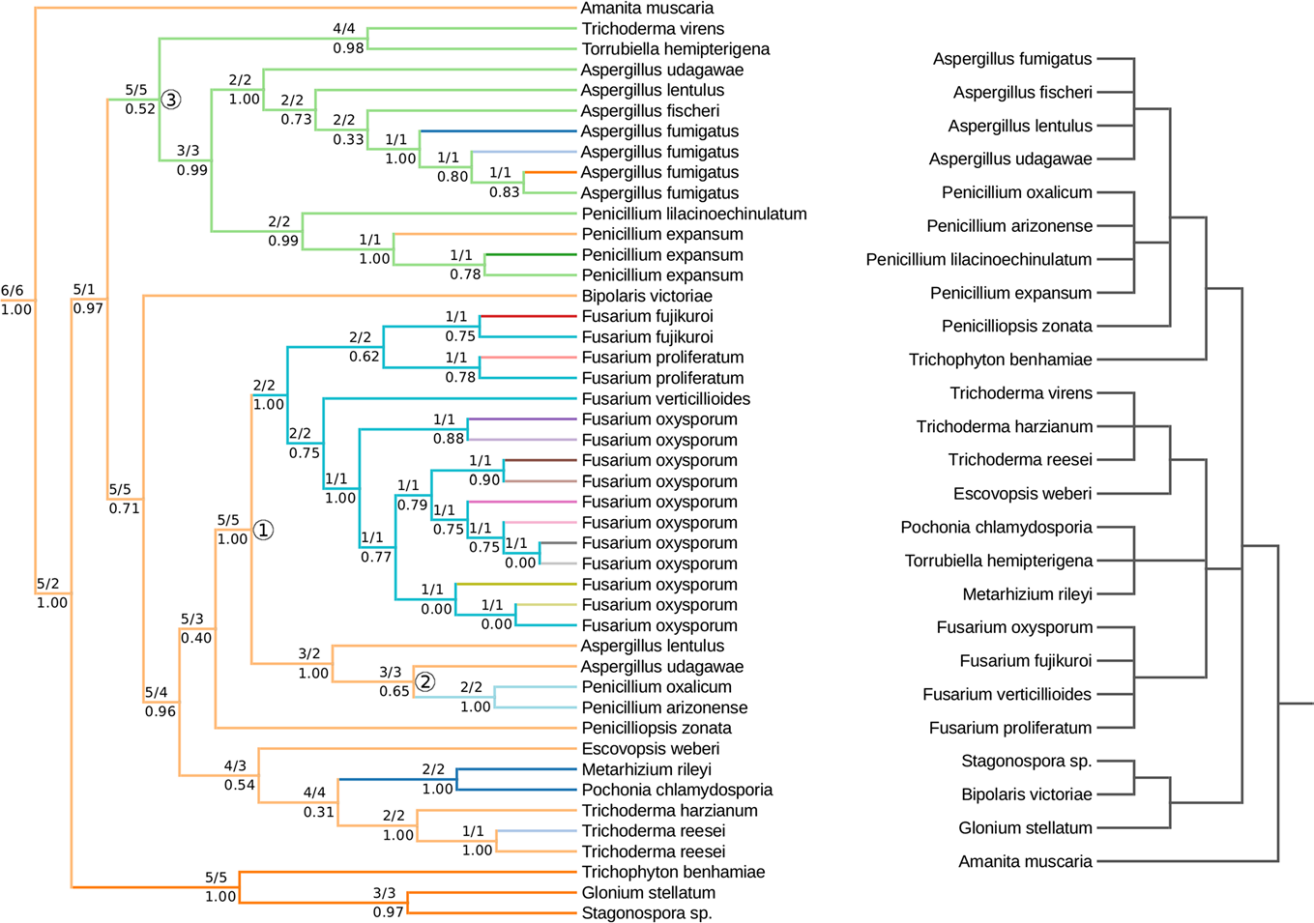
Gene tree of homologs of protein *ABV*48733.1 and species tree retrieved from the NCBI Taxonomy database. Numbers above branches show the values of *I*/*P* mappings before decomposition, numbers below branches show the aLRT support. The separate histories of different loci have been highlited by different colors

The sequences have been aligned using MAFFT and trimmed with TrimAL [30, 31]. The phylogenetic tree has been created using PhyML with default parameters and aLRT branch support, and rooted by setting *Amanita muscaria* as the outgroup [32]. Nodes of the species tree have been collapsed to represent only the following taxonomic ranks: species, genus, family, order, class, phylum, kingdom. The gene tree has been reconciled with NCBI Taxonomy with loss weight 1, duplication weight 1.5, co-divergence weight 0 and transfer weight varying from 3 to 8. The result of decomposition by our heuristic approach has been visualized using the Python ete3 package [33] and is depicted in Fig. 8.

The protein *ABV*48733.1 has been chosen for analysis because it exhibits a particularly complex evolutionary history. In the gene tree consisting of 45 homologs, our heuristic has inferred 23 locus acquisition events. Depending on the transfer weight, Notung 2.9 reported from 1 to 7 transfers and numerous duplications. The inference of evolutionary events is further complicated by the fact that the sets of transfer edges for transfer weights 3 and 6 are disjoint. However, even though in this case it is difficult to explain the whole evolutionary scenario by reconciliation, the decomposition can still be helpful in inferring biologically relevant conclusions.

Consider the light blue subtree on Fig. 8 composed of several *Fusarium* species. The protein has been extensively duplicated in *Fusarium oxysporum*. Furthermore, the light blue subtree branches with *Aspergillus* and *Penicillium* species. As the support of the “junction” node of both subtrees (labeled as 1 on the locus tree) is 1.00, we can assume that this is not due to erroneous gene tree inference. The *Fusarium* species are not present in any other part of the tree, which is indicative of a horizontal gene transfer from the ancestor of *Aspergillus* and closely related *Penicillium* species to the ancestor of *Fusarium* species from the light blue subtree. The horizontal gene transfer hypothesis is further supported by the fact that genus *Aspergillus* is distantly related to *Fusarium*. It is estimated that their ancestors separated 300–500 million years ago [34, 35].

The branching of two distant groups of closely related species in this case is also visible from the values of mappings *I* and *P*, as their values at the junction node 1 are considerably higher than the values at the root of the light blue subtree and its sibling node. This transfer is consistent with reconciliation results for transfer weights greater or equal to 3.7.

The emergence of another light blue subtree, consisting of *Penicillium oxalicum* and *Penicillium arizonense*, could similarly be explained by a duplication or a horizontal gene transfer from an ancestor of *Aspergillus udagawae*. However, *Aspergillus* and *Penicillium* species are fairly closely related, and the support of the junction node (labeled as 2) is only 0.65. A closer inspection shows that performing a nearest neighbor interchange operation resolves the incongruence. This suggests that this locus subtree is an effect of an erroneous tree inference. Reconciliation explains this event by a horizontal gene transfer for transfer weights from 3 to 5, and as an ancestral duplication for greater weights.

Now, consider the light green subtree. This subtree contains several species which are present in its sister subtree (*A. lentulus, A. udagawae*) and numerous closely related species. This is indicative of an ancestral duplication. The duplication hypothesis is further confirmed by the values of mappings *I* and *P*. For the junction node (labeled 3), the mapping *P* is considerably lower than the mapping *I*. This indicates a branching of two large, but closely related groups of organisms. Comparison of mappings *I* and *P* at junction nodes and their children can in future serve as a basis for automated classification of locus gain events as transfers or duplications.

Most other locus gain events are ambiguous, both in the case of history decomposition analysis and the tree reconciliation.

## Conclusions

In this work, we have investigated two new problems, LTI and CLTI, for locus tree inference in a parsimony framework, defined as problems of decomposing a gene tree into trees consistent with a species tree. We have proposed a new mapping, called the *highest separating rank*, which has been applied to improve the classification of duplications and to solve CLTI. We have proposed two memory and time efficient solutions to the proposed problems: an $O(n^2)$ dynamic programming algorithm for LTI and a near linear time heuristic for CLTI designed to solve large instances. Next, to verify the evolutionary consistency of the output our algorithms, we have proposed a validation method based on the model of evolutionary scenarios with HGTs. Finally, we have performed a number of tests on simulated data showing that these algorithms detect evolutionary events with high accuracy, and performed a proof-of-concept analysis of an empirical gene tree. Our results suggest that the new mapping, combined with the lca-mapping, can be used to locate cases of gene duplications and horizontal gene transfers.

### Future outlooks

We plan to extend the solutions to LTI and CLTI to non-binary gene trees, as it would allow to collapse nodes with low support and possibly to decrease the forest size. We will further investigate the properties of the *highest separating rank* mapping, especially in the context of supertree inference, gene tree rooting and gene tree correction. Finally, we plan to apply our methods to design automated tools for HGT inference. They will serve as a preprocessing step in obtaining a manually curated dataset of horizontally transferred genes.
